# Design and Characterization of a Sensorized Microfluidic Cell-Culture System with Electro-Thermal Micro-Pumps and Sensors for Cell Adhesion, Oxygen, and pH on a Glass Chip

**DOI:** 10.3390/bios5030513

**Published:** 2015-07-30

**Authors:** Sebastian M. Bonk, Marco Stubbe, Sebastian M. Buehler, Carsten Tautorat, Werner Baumann, Ernst-Dieter Klinkenberg, Jan Gimsa

**Affiliations:** 1 Chair for Biophysics, Department of Biology, University of Rostock, Gertrudenstr. 11a, 18057 Rostock, Germany; E-Mails: sebastian.bonk@uni-rostock.de (S.M.B.); marco.stubbe@uni-rostock.de (M.S.); carsten.tautorat@uni-rostock.de (C.T.); werner.baumann@uni-rostock.de (W.B.); 2 Leibniz Institute for Farm Animal Biology, Institute of Muscle Biology and Growth, Wilhelm-Stahl-Allee 2, 18196 Dummerstorf, Germany; E-Mail: buehler@fbn-dummerstorf.de; 3 DOT GmbH, Charles-Darwin-Ring 1A, 18059 Rostock, Germany; E-Mail: klinkenberg@dot-coating.de

**Keywords:** lab-on chip, bone cells, thin film platinum sensors, acidification, respiration

## Abstract

We combined a multi-sensor glass-chip with a microfluidic channel grid for the characterization of cellular behavior. The grid was imprinted in poly-dimethyl-siloxane. Mouse-embryonal/fetal calvaria fibroblasts (MC3T3-E1) were used as a model system. Thin-film platinum (Pt) sensors for respiration (amperometric oxygen electrode), acidification (potentiometric pH electrodes) and cell adhesion (interdigitated-electrodes structures, IDES) allowed us to monitor cell-physiological parameters as well as the cell-spreading behavior. Two on-chip electro-thermal micro-pumps (ETμPs) permitted the induction of medium flow in the system, e.g., for medium mixing and drug delivery. The glass-wafer technology ensured the microscopic observability of the on-chip cell culture. Connecting Pt structures were passivated by a 1.2 μm layer of silicon nitride (Si_3_N_4_). Thin Si_3_N_4_ layers (20 nm or 60 nm) were used as the sensitive material of the pH electrodes. These electrodes showed a linear behavior in the pH range from 4 to 9, with a sensitivity of up to 39 mV per pH step. The oxygen sensors were circular Pt electrodes with a sensor area of 78.5 μm^2^. Their sensitivity was 100 pA per 1% oxygen increase in the range from 0% to 21% oxygen (air saturated). Two different IDES geometries with 30- and 50-μm finger spacings showed comparable sensitivities in detecting the proliferation rate of MC3T3 cells. These cells were cultured for 11 days *in vitro* to test the biocompatibility, microfluidics and electric sensors of our system under standard laboratory conditions.

## 1. Introduction

Cell-based *in vitro* systems such as Micro Total Analysis Systems or lab-on-chip systems are commonly used for cell monitoring, cell sorting, or as micro-bioreactors [1,2,3,4]. Chip-based cell-culture systems are a growth market because miniaturization reduces the costs for the systems by reducing the amount of cells and chemical compounds required while enabling the parallelization of investigations in 2D and 3D cell cultures [2,5]. With integrated sensors, the systems can be applied to reduce animal testing in the fields of medical diagnostics or drug development. Using integrated sensors for the *in vitro* measurement, a number of physiological cell parameters can be monitored [6,7,8,9]. For example, parameters such as acidification and respiration have been detected by ion-sensitive field-effect transistors and Clark-type electrodes [10,11,12,13,14,15]. Alterations of the electric impedance of interdigitated electrodes structures (IDES) are measured to detect the initial adhesion, spread, and proliferation of adherent cells [16,17,18,19]. In the future, the number of applications of sensorized cell-culture systems is expected to rise in medical test systems and basic research on cell physiology [2].

Up to now, most of the systems are based on 2D-cell cultures of adherent cells and their monitoring by microscopic techniques and different types of assays, like ELISAs or life-death assays [20,21]. Only a few commercial systems are available for the on-line monitoring of cell physiological parameters. For example, the Bionas^®^ Discovery 2500 system (Bionas GmbH, Rostock, Germany) permits the non-invasive measurement of three metabolic parameters (pH, O_2_, adhesion/proliferation) [22,23,24,25]. Nevertheless, the silicon-sensor technology used in this system is relatively costly and its opaqueness limits the applicability of silicon as a sensor substrate in biological applications.

In this paper, a glass substrate was chosen to permit microscopic observation of the cell culture. The glass substrate carried platinum (Pt) structures, which were covered by Si_3_N_4_ in most chip areas. Bare Pt structures were used for IDES for cell-proliferation monitoring and amperometric oxygen sensors for the registration of the oxygen consumption. Potentiometric pH-sensors were covered with thin Si_3_N_4_ layers as sensitive substrates.

## 2. Experimental Section

### 2.1. Fluidic Structures

**Figure 1:**
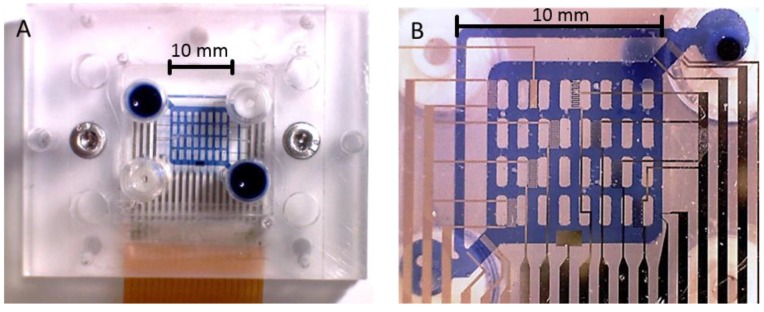
Image of the sensorized cell-culture system. For better visibility, the microfluidic channels were filled with trypan-blue solution; (**A**) Top view. Two screws (silver in color) were used to fix the glass chip with the Pt sensor and ETµP structures in between the PMMA cover and ground plates and to seal the PDMS channels. For the photo, two of the four 1/4 inch 28 microfluidic connectors (top right, bottom left) were sealed with PDMS plugs. The other two connectors (blue) were disconnected for the photo. Flexible circuit-board connector (bottom, brown) was soldered to the chip pads. (**B**) Bottom view through the assembled system.

**Figure 2:**
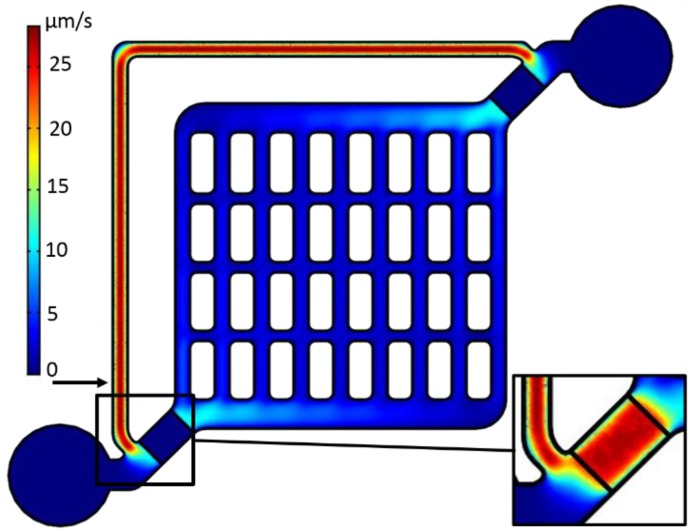
3D-COMSOL^®^ simulations of the velocity distribution in the center plane of the microfluidic structure with sealed-off medium inlets and outlets (lower left and upper right circular structures). The velocity field was calculated assuming a volume force of 31.1 N·m^−3^ induced in the ETµP volume, which was confined by the pump-field electrodes (see Figure 1B and Figure 3). Arrow: site of tracer-particle measurements in the flow-return path. Insert: Velocity profile in the center plane of the ETµP, 125 µm above the chip.

The wall and channel designs of the microfluidic structure were inspired by the general geometry of the Haversian bone-canal system which contains the blood vessels [26]. In the bone, the system ensures a homogeneous distribution of the blood flow and an optimal supply of the native bone-cells, which were also required in our fluidic system. The original channel dimensions were derived in allusion to the geometry of Haversian canals, with an average diameter of 100 µm. Nevertheless, the diameter was increased to avoid clogging by cell growths following observation with 100-µm channels after seven days (J.B. Nebe, personal communication; see also [27]). Owing to the geometry of our sensors, a channel height of 500 µm was used to obtain a roughly quadratic cross-section of the microfluidic channels. The channel widths were 400 µm for the vertical and 275 µm for the horizontal channels (Figure 1 and Figure 2). The peripheral, horizontal supply channels were 800 µm and the separate flow-return path was 300 µm wide. The diagonal inlet and outlet channels were 900 µm wide with a reduced height of 250 µm above the electro-thermal micro-pump (ETµP) structures. This channel-geometry pattern was the result of an optimization process of experimental and 3D-COMSOL^®^ (COMSOL Multiphysics, COMSOL AB, Stockholm, Sweden) simulations (Figure 2). It ensures an even medium supply and cell distribution during seeding, as well as an optimal spreading of the cells in the system.

### 2.2. Fluidic and Electric Connections

The external body of the system consisted of plane, a 6-mm thick ground plate of poly-methyl-methacrylate (PMMA; Plexiglas^®^ GS, Acrylics Deutschland GmbH, Niederfischbach, Germany) and an 8‑mm thick PMMA cover block, which was produced with a micro-milling machine (CCD/2, Bungard Elektronik GmbH & Co. KG, Wndeck, Germany). The two parts were assembled with two M3 stainless steel screws (Figure 1). The cover block carried two 1/4 inch 28 microfluidic connectors for tubes for cell seeding and medium exchange at the top side. The microfluidic structure was imprinted on a poly-dimethyl-siloxane (PDMS) layer located at the bottom of the PMMA cover block. The molds for the structure were produced by stereolithography with UV-curable polymer (RenShape^®^ SL7545, Huntsman GmbH, Switzerland, www.huntsman.com). In order to enhance adhesion, the cover block was coated with bonding agent (GRUN G790, Wacker Silicones, www.wacker.com) before PDMS molding. The microfluidic channels were finally sealed by the pressure between the PDMS layer and the chip surface.

A flexible circuit-board connector was soldered to the glass chip pads (Figure 1A). It protruded between the cover and the PMMA-ground plate, and carried electric dual-inline-pin connectors at its periphery. All surfaces of the chip as well as the microfluidic structure were inert against aqueous solutions, ethanol, and Tergazym^®^ (Alconox Inc., White Plains, NY, USA) and provided the robustness and biocompatibility required for cell culture [28,29,30,31,32]. The cell-culture system was assembled in a laminar flow hood under sterile conditions and operated inside a simple incubator under normal atmospheric conditions at 37 °C. 1/16 inch Teflon (PTFE) tubes were used to connect the system’s inlet and outlet to a medium flask and an external peristaltic pump (Ismatec, IPC-N-4, IDEX Health & Science GmbH, Wertheim-Mondfeld, Germany), respectively. The medium flask was positioned at an elevated position and aerated through a sterile filter. The pump outlet was connected to a waste bottle. The maintenance of a germ-reduced environment was relatively simple because the whole system was hosted by the incubator.

The flow characteristics of the final structure are given in Figure 2. The structures of the master molds were designed with AutoCAD 2010 (Autodesk^®^ Inc., San Rafael, CA, USA) and produced with UV-curable polymer (RenShape^®^ SL7545, Huntsman GmbH, Switzerland) by stereolithography. The microfluidic PDMS structures were cast from the master molds using a 10:1 (v/v) mixture of PDMS prepolymer and curing agent (Sylgard 184, Dow Corning Co., UK) after debubbling of the PDMS in vacuum. The PDMS mold was cured at 100 °C for 1 h and fixed topside up to the molding cover of the MicCell system (GeSiM GmbH, Grosserkmannsdorf, Germany) which served as the carrier of the microfluidic channel system.

### 2.3. The on Chip Structures

**Figure 3:**
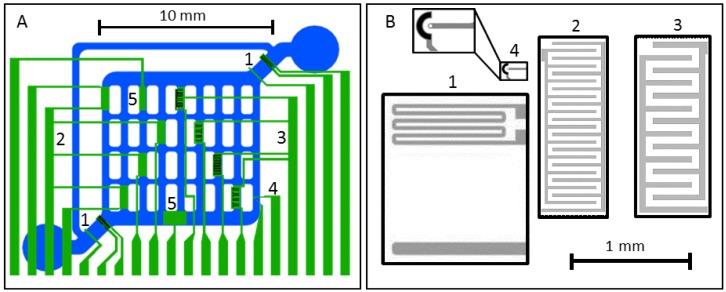
Scheme of the fluidic channels (blue) and location of the Pt-sensor structures (green) on the glass chip: (**A**) ETµP (1), eight common connectors for IDES with 30-µm (2; four connectors from left) and 50-µm (3; four connectors from right) finger widths, one oxygen (4) and two pH sensors with different sensor areas (5); (**B**) to-scale comparison of IDES (2, 3), directly heated ETµPs (1) and oxygen-electrode structures (4, top left: zoom of structure). The pumping force of the ETµP (1) is generated in the volume confined by the field electrode (lower horizontal stripe) and the common ground for the field electrode and the heating element (lower connector of the meander). Active surfaces of the oxygen sensor are marked in white (working electrode) and black (counter-electrode); finger widths and spacings were equal in each of the two IDES types. The outer boxes (30-µm structures: 1.4 × 0.5 mm^2^; 50-µm structures: 1.4 × 0.54 mm^2^) frame the passivation-free areas.

The chip was designed using AutoCAD 2010. It features two ETµPs, four IDES with 30-µm and four with 50-µm finger spacings, one oxygen sensor, and two pH-sensors (Figure 3A). For each 4-inch glass wafer, eight 22 mm × 27 mm chips were photolithographically processed in a lift-off process using chrome masks (GeSiM GmbH, Grosserkmannsdorf, Germany). All the sensors and on-chip connections were Pt structures with a thickness of 100 nm. Most of the chip surface was passivated with a 1.2 µm thick Si_3_N_4_ layer.

### 2.4. ETµP

Two directly heated ETµPs were located in the 900 µm wide inlet and outlet channels. Width and length of the ETµP structures were adapted to fit these channels (Figure 3). The channel height was reduced to 250 µm above the ETµP structures. This was necessary because of the limited effective reach of the temperature and electric pump fields [33]. Details of the pump designs are given in [33,34,35,36]. The 100-µm wide field electrode was not passivated, in order to obtain a large frequency plateau of the pump velocity by avoiding the frequency-dependent capacitive bridging of the passivation layer [36]. Also, the heating meander was not passivated because it serves as the field ground electrode in the directly heated ETµP design. The heating-meander structure consisted of six parallel 30 µm wide and 950 µm long conducting paths of the Pt layer. The distance of the field electrode to the heating meander was 800 µm (Figure 3B). The measurements were conducted with a DC heating voltage of 4.29 V applied to the heating meander and an AC field-electrode voltage between 20 V_rms_ and 45 V_rms_ for selected frequencies in the frequency range between 100 kHz and 10 MHz. The conductivity of deionized water was adjusted to 0.1 S/m by adding NaCl. The flow velocity induced by the ETµP was measured microscopically with tracer particles (800-nm latex particles) in the 300-µm wide flow-return path (Figure 2).

### 2.5. Interdigitated Electrodes Structures (IDES)

Based on our experience with preceding systems, IDES with two different pitches were designed to consider the influence of the scaling on the sensitivity of cell proliferation and the degree of cell adhesion detection [17,19]. Pitches (electrode and gap widths) of 50 µm and 30 µm were chosen (Figure 3B). Because of their roughly equal overall electrode areas, the 30-µm IDES had more fingers and inter-electrode gaps than the 50-µm IDES. We assumed that a lower pitch would lead to an increased IDES sensitivity, especially for smaller cell species. Smaller cells should be better able to cover larger percental parts of the IDES fingers and to bridge the electrode gaps. COMSOL^®^ simulations showed an enhanced IDES field strength for the lower pitch, which could improve the sensitivity of the detection of smaller cells. The rectangular-shaped passivation-free areas above the 50-µm and 30-µm IDES were 0.75 mm^2^ and 0.70 mm^2^, respectively, while their non-passivated electrode-metal areas were 0.37 mm^2^ and 0.34 mm^2^ (Figure 3B).

An impedance/gain phase analyzer (HP4194A, Agilent Technologies, Inc., Santa Clara, CA, USA) was used to follow the IDES-impedance changes during proliferation of the MC3T3 cells. Cell proliferation was followed microscopically over 11 days to check the cell status and distribution on the different IDES in parallel. The impedance data was recorded in pairs of the real and imaginary parts over a frequency range from 100 Hz to 1 MHz. A simple RC model, *i.e.*, a parallel circuit of a resistor (R) and a capacitor (C), was applied to each data pair to obtain frequency-dependent resistance (R(f)) and capacitance (C(f)) values. As already described (Koester *et al.*, 2010), only the capacitance values (C(f)) were used for further analysis. A C(f)-reference spectrum (C_PDL_) was determined after PDL coating. It was used to standardize the C(f) spectra during cell proliferation (C_cell_). ΔC-peak values (C_PK_) were directly obtained from the data points of the standardized spectra –ΔC = C_PDL_ − C_cell_. Plots of the C_PK_ values over days *in vitro* describe the cell-proliferation behavior. The time dependencies of the C_PK_ values were finally fitted by a modified Verhulst-Pearl equation [37]:

(1) $$C_{PK}\left(t\right)=\frac{C_{PK}^{min}}{\frac{C_{PK}^{min}}{C_{PK}^{max}}+\left(1-\frac{C_{PK}^{min}}{C_{PK}^{max}}\right)e^{\left(\frac{-\ln\left(2\right)}{T}t\right)}}$$

where $C_{PK}^{min}$, $C_{PK}^{max}$, *T*, and *t* stand for the smallest and highest *C_PK_* value, the cell-doubling time and time in days *in vitro*, respectively.

### 2.6. Oxygen Electrodes

The oxygen sensor consisted of a circular working electrode with an area of 78 µm^2^ and an engulfing auxiliary electrode of 1650 µm^2^ in 20 µm distance to the working electrode (Figure 3B, structure 4). An external flow-through Ag/AgCl microelectrode (Microelectrode Inc., Bedford, NH, USA) in the outlet tube was used as reference electrode.

The design resembles that of a Clark-type amperometric sensor, though without an oxygen-selective membrane. We checked that the sensor could be used without the membrane under defined medium conditions. Amperometric and cyclic voltammetry measurements were performed with a PalmSens potentiostat (PalmSens BV, Utrecht, The Netherlands).

### 2.7. pH Electrodes

To simplify the wafer processing, we tested Si_3_N_4_ for its pH sensitivity. Test sensors were produced, starting from microscopic glass slides with a Pt coating. After depositing a 20 nm or 60 nm thick layers of Si_3_N_4_ by plasma-enhanced chemical vapor deposition, the layers were sealed by approximately 300 µm PDMS before round openings with an area of 3.14 mm^2^ were introduced. To soak the Si_3_N_4_ layers, the sensors were washed with deionized water and stored overnight in phosphate-buffered saline (PBS, Biochrom AG, Berlin, Germany) [38].The sensor and the external Ag/AgCl-reference electrodes were exposed to test solutions of different pH (PBS titrated with HCL or NaOH) and the potential was measured with the PalmSens potentiostat. For characterization, the pH was cycled, sweeping the range from 6 to 8 in integer-pH steps. The measured voltages were checked for their reproducibility and possible hysteresis effects. Three sensors were measured for each Si_3_N_4_-layer thickness.

After the pH sensitivity of the Si_3_N_4_ layers was confirmed, pH sensors of the two different sizes were integrated into the chip. Their working windows with 20 nm or 60 nm thick sensor layers of Si_3_N_4_ were framed by the 1.2 µm thick Si_3_N_4_-passivation layer. We tested that smaller on-chip sensors with 0.5 mm^2^ did not show a higher noise magnitude and their sensitivity was comparable to the test sensors at a slightly faster response time. For these reasons, the larger sensors with 1 mm^2^, which did not fit to the 400 µm channels, were not further considered (Figure 3A, structures 5).

### 2.8. Cell Culture

Mouse-embryonal/fetal calvaria fibroblasts (MC3T3-E1) were obtained from the German collection of microorganisms and cell culture (DSMZ, Braunschweig, Germany). The cells were cultured at 37 °C in 50 mL cell-culture flasks (25 cm^2^; Greiner Bio-one, Frickenhausen, Germany) in Alpha medium (ord. No. F 0925) [26,27,39]. This is a modified Eagle’s medium (MEM) supplemented with 1% penicillin/streptomycin (stem solution: 10,000 U/mL penicillin/10,000 µg/mL streptomycin) and 10% fetal bovine serum (all purchased from Biochrom AG, Berlin, Germany). The incubator ensured 95% humidity in a 5% CO_2_ atmosphere. The complete medium was replaced every other day. Cells grown to confluence were trypsinated (0.05% Trypsin + EDTA 0.02%, PAN Biotech GmbH, Aidenbach, Germany) and resuspended before subculture, transfer to the microfluidic system or separate IDES measurements.

For cell culture, the microfluidic system was cleaned with an aqueous 70% ethanol solution and deionized water before being autoclaved at 101 °C for 1 h. The tubes, connectors, chips, and medium bottles were autoclaved at 120 °C for 30 min.

**First experiment (long term cell culture in the confined microfluidic system):** Because CO_2_ buffering was not possible in the closed microfluidic setup, carbonate-free Alpha medium (P03-2510, Pan Biotech, Aidenbach, Germany) was used and buffered by the addition of 60 mM HEPES. The microfluidic channels were not coated. The cell-number of the seeding suspension was adjusted to 50,000 cells per mL with a Neubauer cell-counting chamber, corresponding to approx. 1750 cells for the 35 µL volume of the system (the overall bottom surface of the channels was approx. 70 mm^2^; compare to Figure 1B). The microfluidic system was flushed with buffered medium before the cell suspension was injected under a sterile hood. After this, the systems were transferred to the incubator and sealed or connected to a medium flask and a peristaltic pump located between the system and waste flask. The external pump velocity was adjusted to 1 µL/min. The medium flask was exchanged every third day. For the sealed systems, the medium exchange was conducted with a syringe under a sterile hood every other day.

**Second experiment (IDES detection of cell proliferation):** For separate measurements with the IDES cell-adhesion sensors, a round plastic trough (inner diameter 14.4 mm) was glued with biocompatible glue MED-1511 (NuSil Silicone Technology, Carpinteria, CA, USA) to the chip confining the cell suspension to the chip’s sensor area (compare to Figure 3A). The chips were coated with 200 µL of 100 µg/mL poly-D-lysine (PDL, poly-D-lysine hydrobromide, Sigma Aldrich, Taufkirchen, Germany) for 4 h to enhance cell attachment [40]. After removing the PDL solution the cells were seeded on the chip at a concentration of 250,000 cells/mL, corresponding to approx. 155 cells/mm^2^ (approx. 25,000 cells per chip) and cultured as described above. The chips were transferred to the incubator and the cells were allowed to adhere for 24 h before the first impedance measurement. The culture medium (Alpha-medium) was exchanged daily, immediately before the measurements.

**Third experiment (detection of cellular respiration):** For testing the oxygen sensors, the microfluidic systems were prepared as described for the 1st experiment. In order to swiftly obtain strong sensor signals 1.5–1.6 million cells per mL (approx. 50,000 cells per microfluidic system) were seeded.

**Fourth experiment (detection of cellular acidification):** The pH sensors were tested under the same conditions as the oxygen sensors, except that unbuffered medium was used for pH recordings.

## 3. Results and Discussion

### 3.1. Cell Culture in the Microfluidic Structure

As a first test of the microfluidic system, cell growth was investigated in flow-free systems with sealed inlets and outlets. Under these conditions, stable cell growth was observed for two days before the phenol-red indicator of the medium indicated a significant pH change. Nevertheless, the necessary medium exchange with a syringe was not routinely possible. Bubble injection and contamination usually led to strong distortions of the cell culture.

A clear improvement was obtained with self-contained, microfluidic systems in a standard incubator. Their continuous perfusion allowed for cell culturing without contamination for up to seven days. The limiting factor was the accumulation and growth of gas bubbles in the system if they could not be flushed out by the flow of medium. The bubbles could dispel or even lead to cell death.

### 3.2. ETµPs

**Figure 4:**
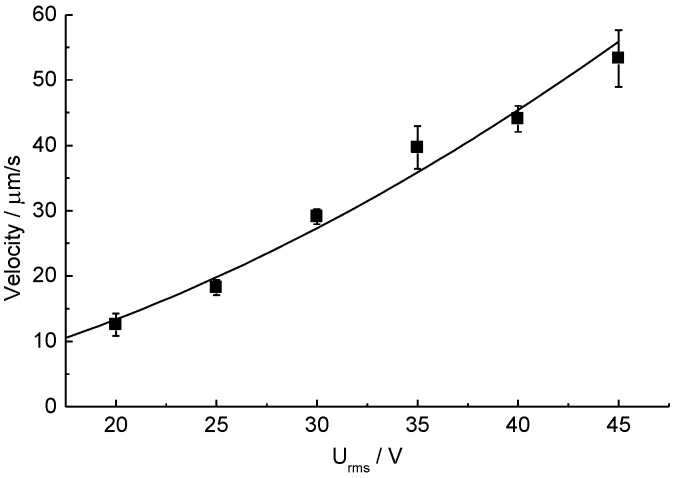
Pumping velocities induced in the flow-return path of the sensorized cell-culture system. Each data point represents the means of 10 tracer particles near the center of the path.

Only one ETµP was activated to characterize the flow properties of the system (Figure 4). Theoretically, the second pump would not increase the flow velocity but, instead, the available pressure [41]. This would not significantly increase the flow velocity in our microfluidic structure without cells, because the friction is relatively low in this case. The pump velocity was measured with tracer particles (800 nm latex) in the flow-return path (arrow in Figure 2). Figure 4 shows the dependence of the velocity on the field-electrode voltage $U_{rms}$ for a field-electrode frequency of 1 MHz and a DC-heating voltage of 4.29 V at a medium conductivity of 0.1 S/m. Fitting the velocity *v* with:

(2) $$v=aU_{rms}^{b}$$

to the measuring data provided parameters of a = 0.067 ± 0.034 µm·s^−1^·V^−b^ and b = 1.77 ± 0.15 for *R*^2^ = 0.968 [33]. The pump velocity could easily be adjusted either by varying the field-electrode or the heating-element voltages [35]. No bubbles are generated by the ETµPs at the working frequency of 1 MHz. Further we checked that the ETµPs could be operated at conductivities >1 S/m and with cell-culture media [33,36].

### 3.3. Interdigitated Electrodes (IDES)

**Figure 5:**
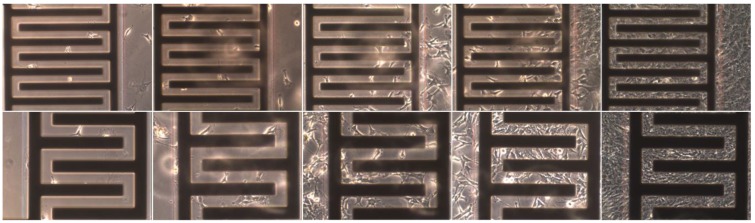
Microscopic images series of cells growing on 30-µm (**Top**) and 50-µm (**Bottom**) IDES in the center of the chip. The images were taken on days 1, 3, 5, 7, and 9 (compare to Figure 6).

Classical cell culture in an open trough was used to simplify the IDES characterization experiments. Figure 5 presents two series of microscopic images of cells growing on IDES with two different finger pitches. The measurements were conducted for 11 days.

**Figure 6:**
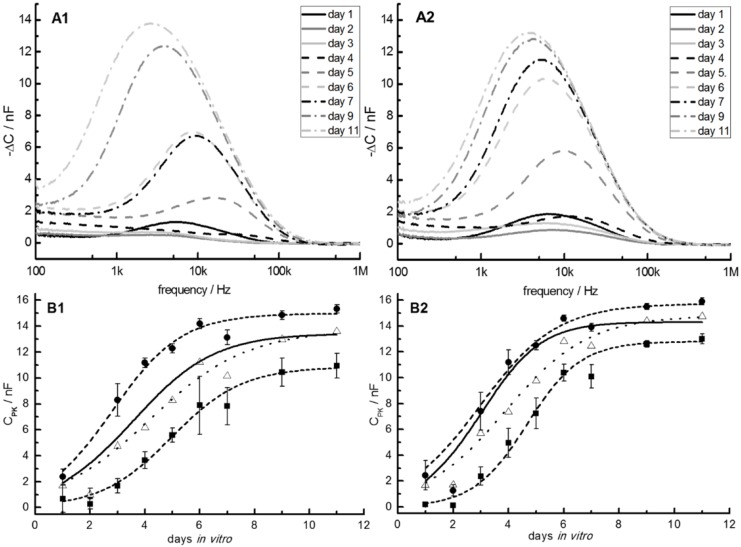
(**A1**,**A2**): Typical examples for the frequency dependencies of the capacitance differences (−∆C) between the IDES capacitance during cell proliferation and the cell-free reference of five chips with four 30-µm IDES (**A1**) and four 50-µm IDES (**A**2) over 11 days of cell growth. (**B1**,**B2**): For each chip, the C_PK_ values (*i.e.*, the –ΔC-peak values, see text) of the spectra were averaged for the four 30-µm (**B1**) and four 50-µm (**B2**) IDES. For comparison, curves for the highest (dashed line, circles) and lowest (dashed line, squares) averaged C_PK_ values are shown, reflecting the limits of the cell-proliferation rates observed. From Verhulst-Pearl fits, the parameters in columns A of Table 1 were obtained when the data from all the chips were pooled for a certain IDES pitch (dotted lines). Parameters obtained from averaging the separately fitted parameters of each chip are given in columns B of Table 1 (solid lines). The experimental points for day 2 were not considered in the fits (see text).

During cell proliferation, the −ΔC spectra of both IDES-sensor geometries changed and their peak heights largely correlated with the number of cells on the sensors (Figure 6A). Plots of the fitted peak values, C_PK_, over proliferation time showed a sigmoidal shape, which could be fitted with Equation (1) (Figure 6B) [42]. Characteristically, a C_PK_ decrease was observed in the initial phase of cell proliferation at day 2. One possible reason may be that the cells were synchronously massing together at the end of the lag-plateau phase as part of the cell cycle. Because of this particular behavior, the C_PK_ data on day 2 was not considered in the fits. Beyond these points, differences in the C_PK_ were caused by changes in the cell number and cell properties during proliferation, rather than by the different IDES geometries. A slightly larger variation was found for the 30-µm IDES (Figure 6).

Nevertheless, the results with the 30-µm and 50-µm IDES of five chips were very similar with respect to sensitivity, the shapes of their −ΔC spectra and the C_PK_ plots-over-proliferation times. A comparison of the averaged C_PK_ values for 11 days *in vitro* showed slightly (though still significantly) higher values (Mann-Whitney rank-sum test; *p* < 0.05) for the 50-µm IDES (13.89 nF ± 1.31 nF) compared to the 30-µm IDES (13.29 nF ± 1.67 nF) for the confluent cell layers. We checked that the plateau differences were not caused by the sensor distribution on the chips. For example, one of the 30-µm IDES (upper left sensor in Figure 3A) was separated from the axially symmetric distribution of the other sensors. After cell seeding in the centre of the trough, this sensor showed a retarded signal increase. Only after four days *in vitro*, the C_PK_-values rapidly approached those of the other 30-µm sensors. We attributed the slower initial increase to an uneven cell distribution after seeding.

We believe that the variation between the 30-µm and 50-µm IDES was not caused by a direct electric field effect. During the short measuring periods (less than five minutes per IDES per day), the field strength above the 30-µm IDES was approx. 5/3 higher than above the 50-µm IDES. Nevertheless, clarification of this problem will require further experiments with IDES that had more diverse geometries.

For data interpretation by Equation (1), the *C_PK_* values of each chip were averaged for the four 30-µm and four 50-µm IDES and plotted over days *in vitro*. The values of each of the five chips were fitted by Equation (1) to calculate specific parameters (cell doubling time, $C_{PK}^{min}$, and $C_{PK}^{max}$) for each chip and IDES structure (compare to the limiting curves in Figure 6B). In a second step, the parameters obtained were averaged for the two IDES structures (Table 1, columns B). This method of data refinement was compared to the “common” approach, *i.e.*, separate pooling of the 50-µm and 30-µm IDES data on all chips before fitting (Equation (1); Table 1, columns A).

**Table 1:** Fitted parameters of Equation (1) obtained from pooling the data of a certain IDES pitch of all chips (columns A; dotted lines in Figure 5B) or averaging the separately fitted parameters of each chip (columns B, solid lines in Figure 6B). Please compare to T values of 24 to 48 h given by the DSMZ.

	50-µm IDES				30-µm IDES			
	A		B		A		B	
$C_{PK}^{min}\;$[nF]	0.93	±1.09	0.61	±0.51	1.14	±1.60	0.94	±0.65
$C_{PK}^{max}\;$[nF]	14.77	±2.03	13.89	±1.31	13.69	±3.43	13.29	±1.67
T [d]	1.00	±0.28	0.69	±0.18	1.21	±0.47	0.97	±0.19

For both IDES structures, the pooling of all the chip data led to longer doubling times and higher standard deviations than when averaging the parameters obtained from separate fits of chips. The reason is the variation in the individual C_PK_ behavior. While some IDES showed a rapid C_PK_ increase after cell seeding, others exhibited a longer lag phase. With respect to Equation (1), this can be interpreted as a shift of the abscissa, *i.e.*, different cell proliferation start times. Pooling such data leads to a smeared average behavior and a slope of the averaged C_PK_ increase that is reduced with respect to the individual C_PK_ slopes. The reason for the variability in the shapes of the C_PK_ curves is probably caused by the relatively small areas of the IDES with respect to the whole chip area. Their accessibility during cell seeding depends on the IDES location within the microfluidic structure. This, in combination with random cell sedimentation, will lead to large variations in the cell number on the individual IDES surfaces after cell seeding.

This may lead to three limiting scenarios for cell proliferation: (i) in an ideal case, only one or very few cells attach to an IDES. Their proliferation may then lead to an ideal Verhulst-Pearl behavior with *T* representing the actual cell-doubling time; (ii) high cell numbers on the IDES would ideally resemble the situation of the first case at *t = τ* after multiple cell divisions. Accordingly, the same parameters would be obtained from Equation (1) by shifting the abscissa, *i.e.*, substituting *t* for *t’* *=* *t* *+* *τ*; (iii) in a third limiting case, no cell would adhere to the IDES after seeding. Cells would grow to confluence in the vicinity of the IDES before they start overgrowing the IDES with one or more moving fronts. This may also lead to a sigmoidal-like *C_PK_* behavior, though with a long lag phase followed by a rapid growth to confluence. Equation (1) would not apply.

Most probably, the issue of detecting cell-doubling times would be better defined for IDES with large detection areas, ideally for a single IDES covering the whole surface of the chip. Nevertheless, distributed IDES help resolve the growth patterns on the cell-chip surfaces.

Moreover, various physiological cell properties will modulate the detected *C_PK_* behavior. After cell seeding, the largest effects will stem from the number of cells adhering to the IDES, cell size and shape. The consequent spreading and proliferation will be influenced by the surface-coat properties (here the PDL layer) and the ability of the cells to secrete native adhesion molecules. These molecules condition the culture medium and improve cell adhesion, which increases the proliferation rate and possibly the end-plateau levels. The *C_PK_* over days-*in vitro* plots of Figure 5B suggest a correlation of steeper slopes with higher plateau levels. Even though we could not generally confirm this correlation, our results suggest that differences in the density of the PDL layers influence the proliferation-slopes as well as the plateau-levels of the sigmoidal Verhulst-Pearl fits.

In the microfluidic cell-culture system, the IDES cell-proliferation measurements were comparable to the above experiments up to four days. Within this time, maximum *C_PK_* values of approx. 5 nF were reached. Nevertheless, when the culture was continued for a week (see “cell culture in the microfluidic structure”) the *C_PK_* values did not further increase and started fluctuating around values lower than 5 nF. Our observations suggest that cell adhesion was hindered in the vicinity of the contact zones of the PDMS walls and the glass chip. 

The *C_PK_* values were correlated to the cell numbers which were microscopically observed at a certain IDES (compare to Figure 5). Generally, lower cell numbers were found at IDES in microfluidic channels with higher flow velocities (Figure 2). In areas of low flow velocity bubble formation was more pronounced. In most cases, bubble formation was the reason for the termination of a certain measurement before seven days of culture.

### 3.4. Oxygen Sensors

**Figure 7:**
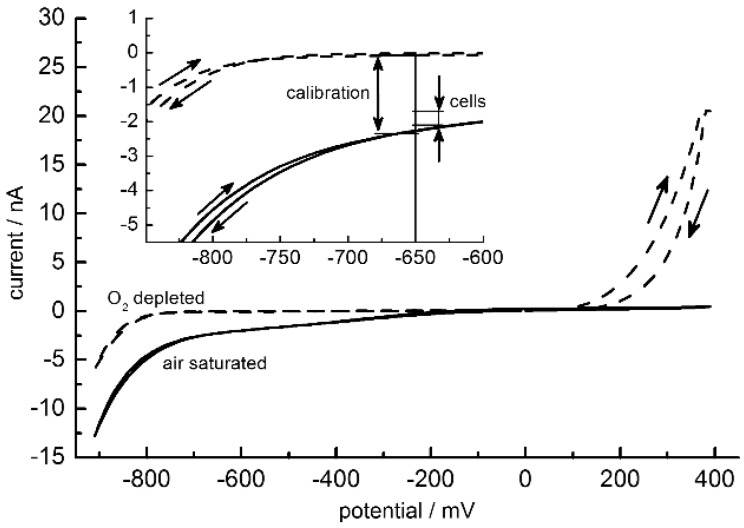
Mean of 10 cyclic voltammograms of a 25-µm oxygen electrode in two-electrode configuration between −900 and 400 mV (step potential 10 mV, scan rate 10 mV/s) for air-saturated (solid lines) and oxygen-free media (dashed lines). Voltammetric scan directions are marked by arrows. Insert: zoom around −650 mV. Double arrows mark the current ranges swept during calibration of the electrode (−2.346 ± 0.038 nA (oxygen saturated); −0.073 ± 0.007 nA (oxygen depleted)) and during cell culture at −650 mV (Figure 8). Please note that the steep current increase for the oxygen-free medium above 100 mV is induced by the presence of 1% sodium sulphite.

The signals of amperometric oxygen sensors are known to depend linearly on the oxygen concentration [43]. This allowed for a two point calibration by air-saturated (21% oxygen) and oxygen-depleted medium. Oxygen depletion was achieved by the application of 1% sodium sulphite. To characterize the sensors under Alpha medium conditions, cyclic voltammetry was conducted in a three-electrode setup with the Ag/AgCl reference electrode in a small beaker. The aim was to find an optimal working potential that combined a high sensitivity with a good discrimination against possible electrochemical distortions by medium compounds and electrode processes. Distortions by redox-active substances were negligible in the cyclic voltammograms except for a steep current increase in the oxygen-free medium above 100 mV (Figure 7). The increase was induced by the presence of sodium sulphite. In order to improve the signal-to-noise ratio of the sensor in cell-culture measurements, the diameter of the working electrode was increased from 10 µm (Figure 3B, structure 4) to 25 µm (not shown).

For the operation of this sensor in the cell-culture medium, a working potential of −650 mV was chosen instead of −700 mV as used in commercial systems. This ensured a better discrimination against alterations of the electrode properties by electrochemical processes around −700 mV (compare to Figure 6). Such processes had a stronger effect at the steeper slopes of the current-potential curves below −750 mV.

With the three-electrode setup, a current difference of 2.21 ± 0.3 nA was obtained between the air-saturated and oxygen‑depleted media at −650 mV (results not shown). Nevertheless, in the microfluidic system, the working electrodes were rapidly deteriorating when gas bubbles in the channel screened the reference electrode from the working and counter electrodes. In such cases, the current was electronically up-regulated in order to keep the detected working potential at −650 mV. To overcome this problem and to protect the working electrode from the current up‑regulation, a simpler two-electrode configuration was tested in which the counter and reference electrodes were joined in a single external electrode and operated against the on chip-working electrode. With this setup, from cyclic voltammetry measurements a signal difference of 2.23 ± 0.11 nA was obtained between the air-saturated and oxygen depleted media at −650 mV (Figure 7). A pH dependence could not be detected in cyclic voltammetry measurements in the pH range from 6 to 8.

To calibrate the microfluidic setup under cell-culture conditions amperometric measurements were conducted at −650 mV and 37 °C. Calibration with air-saturated and oxygen‑depleted media yielded a current difference of 2.27 ± 0.04 nA which is very similar to that found in cyclic voltammetry measurements (please compare current differences of cyclic voltammetry and calibration along the light vertical line in the zoom in Figure 7).

Before cell-culture experiments, the oxygen sensor of each chip was calibrated in the closed microfluidic system for more than five hours with oxygen-free cell‑culture medium, followed by rinsing and calibration with air-saturated medium for more than 16 h. As in cell culture experiments, the current was logged 5 s after electrode activation at a potential of −650 mV *vs.* the external Ag/AgCl electrode. In order to reduce the overall oxygen consumption during cell culture, data was logged only every 20 min.

**Figure 8:**
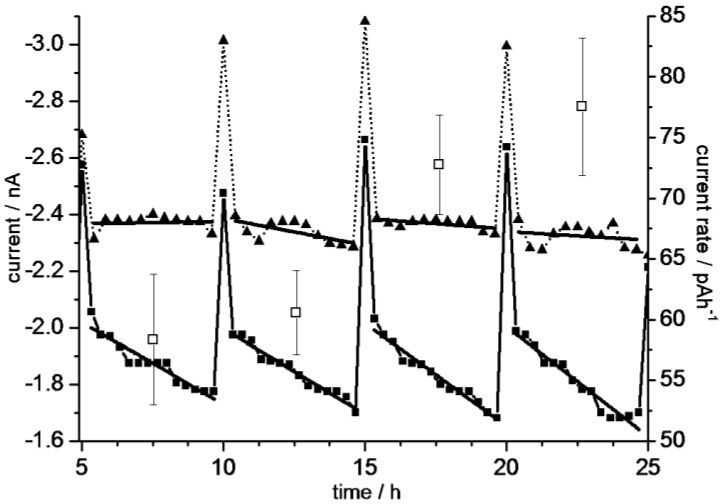
Exemplary oxygen-sensor currents (left ordinate) measured in the microfluidic cell-culture system without cells (dotted lines, triangles) and in the presence of cells (solid lines, squares). The five-hourly current peaks correspond to the pumping cycles of medium exchange during cell culture. The abscissa data without cells was normalized to correspond to the measurements with cells. Measuring points in between the pump cycles were fitted by lines. The continuously increasing slopes in cell culture describe current rates which correspond to increasing oxygen-consumption rates of cells (right ordinate, open squares). The minimum and maximum of all lines with cells are plotted in the insert of Figure 7, marked by “cells”.

In cell-culture experiments, the medium in the microfluidic system was fully renewed every 5 h by an external peristaltic pump. The slight risk of cell detachment at pump rates of 300 µL/min for 5 min was significantly reduced in later experiments when the medium was exchanged at a rate of 150 µL/min for 6 min. Figure 8 presents measurements starting four hours after cell seeding. After approx. 29 h the cells reached confluence, *i.e.*, the increase in the current rate detected over 25 h (right ordinate in Figure 7) is correlated to the microscopically observed increase in cell number.

Using the exemplary data of Figure 8, the current-sweep range in the cell culture corresponded to oxygen concentrations between 88% and 72% with respect to 100% air saturation at 37 °C. This suggests that the cells were cultured near to O_2_-saturation in our microfluidic system. This may be caused by the medium volume per cell which was much higher than in comparable commercial systems [23]. Another contribution may be the high oxygen-diffusion coefficient in PDMS (5.00 × 10^−5^ cm^2^/s at 38 °C); which is even higher than in water (3.33 × 10^−5^ cm^2^/s at 40 °C [44,45]). The averaged current rates detected with and without cells differed by an approx. factor of ten (Figure 8). The sensor sapping was negligible in our measuring mode with one current log per 20 min. We therefore think that our data in cell-free media represents the sensor drift while the current rates under culture conditions largely correspond to the oxygen-consumption rate of the cells. Consequently, we correlate the increase in the current rates over 25 h to an increasing overall respiration due to the observed cell proliferation.

When the cell culture was continued for up to 80 h the detected current rates changes started to fluctuate. The probability of the formation of larger gas bubbles increased. Their removal needed additional flushing in connection with tilting and tapping of the system which probably contributed to the fluctuations.

Despite of its simplified two-electrode setup, the sensitivity of our oxygen sensor was comparable to commercial systems [16,46]. In our system, a more frequent current logging would be possible without significant errors introduced by sensor sapping. 25 µm-Pt sensors could also be used in cell-culture systems with smaller volumes for a more rapid detection of the effects of chemical compounds on the cellular metabolism which would be interesting for animal-replacement methods e.g., in pharmacology (compare to [24,40]).

### 3.5. pH Sensors

To characterize the pH sensors, their potentials were detected against the Ag/AgCl reference electrode between pH 4 and pH 9. A minimum of three test sensors was used for the two sensor-layer thicknesses. All readouts reached 90% of their 600-s end potentials after approximately 60 s. We considered 60 s to be the fast‑response time of the pH-sensors according to the site-binding model of Bousse and Bergveld [30,47].

**Figure 9:**
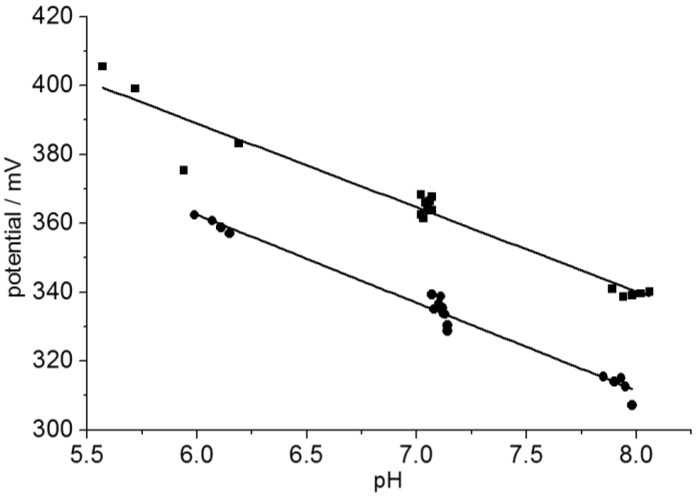
Cyclic potential measurements with a 20-nm (squares) and a 60 nm pH sensor (circles) between pH 6 and pH 8. The potentials were detected after 5 min before solution exchange and plotted over the reference pH. Sensitivities of −24.8 ± 0.8 mV (20 nm) and −25.6 ± 0.9 mV (60 nm) per pH step were obtained from linear fits.

To determine the sensitivity of the sensors and possible hysteresis effects, cyclic measurements were carried out against the Ag/AgCl reference electrode between pH 6 and pH 8 in integer steps by buffer exchange every 600 s. The 600-s sensor potentials were plotted over the reference pH (Figure 9). The electrodes were not rinsed before exchange to resemble the experimental situation in the cell culture. A conventional pH glass electrode (InLab micro-pH electrode, Mettler-Toledo GmbH, Gießen, Germany) was used as a reference. The sensor voltages were recorded continuously. Both the sensor voltage and the glass electrode showed a minute but steady pH decrease during the experiments.

The linear fit slopes were −24.8 ± 0.8 mV for the 20 nm and −25.6 ± 0.9 mV for the 60 nm pH-sensors. In experiments over two pH steps (6, 7, and 8), no hysteresis, *i.e.*, no direction-dependent potentials at pH 7, were found, while hysteresis was observed in experiments spanning five pH steps (4 through 9; results not shown). Such a sweep-range dependent hysteresis has been described by the model of Köhler and Landgraf [31]. In practice, a hysteresis appearing for wider sweep ranges is caused by an incomplete pH equilibration of the sensor substrate [29,30,47].

Naturally, a high sensitivity and reproducibility around the physiological pH of 7.4 will be most important in cell-culture measurements. While a sensor equilibration within less than a minute would be desirable for pharmacological cell testing based on cellular acidification rates [22,23,24], it can be slower in standard cell culture applications.

To check the stability of the sensors, drift rates were determined in long-term experiments at pH 7.4 in a 37 °C-PBS solution for 27 h (results not shown). Typical drifts of 0.27 mV/h and 0.17 mV/h were obtained for the 20- and 60-nm sensor layers, respectively. These values are comparable to those reported by Redlin [38].

Before cell culture, the 60-nm on-chip sensor (Figure 3A) was calibrated with test-buffer solutions of pH 7 and 8 in the microfluidic system. The solutions were introduced into the cell-free, sterile system and the potential was recorded against the external flow-through Ag/AgCl electrode for two hours. Before each measurement, the system was thoroughly rinsed with PBS of pH 7.4. With the fresh sensors, slight drifts of the potentials of approx. −8.16 mV/h were observed for both pH values. Nevertheless, because the drifts were synchronous their influence on the potential difference was negligible. The averaged potential difference was determined from the potentials taken at the times after solution exchange for pH 7 and 8. With −39.28 ± 0.96 mV, the fresh sensors were clearly more sensitive than those used in Figure 8.

**Figure 10:**
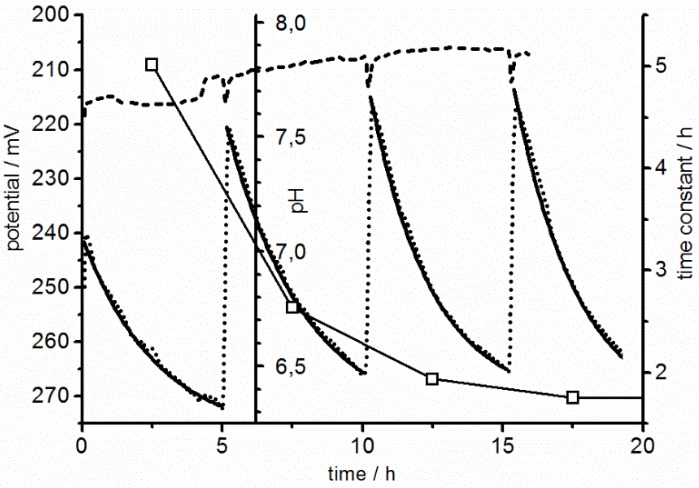
Exemplary measurements with a 60-nm pH sensor in a cell-free chip (reference) and under cell culture conditions. As a reference, three measuring cycles were conducted with cell culture medium of pH 7.8 (dashed line). Medium exchange events can be recognized by glitches every five hours. Using a two-point calibration, a drifting pH scale (shown at 6.2 h) can be obtained from the drifting reference potential at pH 7.8 (see text). With cells a steady acidification was observed after each medium exchange (dotted line). Fits of single exponentials (Equation (3)) led to time constants decreasing for increasing cell numbers (squares, referring to right ordinate).

Figure 10 shows pH recordings in the microfluidic system during cell culture. A relatively high medium pH of 7.8 was chosen to prevent adverse physiological effects by the cellular acidification. Before cell seeding, the system was tested with cell-culture medium for three pump cycles. A drift very similar to that seen with the test-buffer solutions was observed (Figure 10). We therefore think that actual pH values can be recalculated using the average calibration potential difference of −39.28 ± 0.96 mV in combination with a drifting reference at pH 7.8 (compare to drifting pH scale in Figure 10).

To characterize the pH sensors with cells, high cell numbers were seeded in order to reach a confluent cell layer within a short period of time and maximal pH effects induced by the cellular acidification of the unbuffered medium. Non-adherent cells were flushed out in a first pump cycle, before the measurements were started four hours after seeding. The remaining adherent cells formed an almost confluent layer, which was closing up to full confluence during four pump cycles within 20 h. In each pump cycle, the unbuffered medium was fully renewed at a rate of 150 µL/min for six minutes every five hours during the acidification and respiration measurements. In the experiments, the pH decrease was accelerated after each pump cycle, probably also enhanced by the initial recovery of the cells after seeding.

The parameters $pot_{0}$,$\;pot_{\text{∆}}$, and τ standing for the start potential, the potential change, and the time constant of the single exponential function:

(3) $$pot\left(t\right)=pot_{0}+pot_{\text{∆}}\left(1-e^{-\frac{\text{t}}{\tau }}\right)$$

were separately fitted to the measuring pH data between each of the pump cycles in Figure 10. For the fits, the measuring time *t* was set to zero for every measuring cycle. In the literature “acidification rates” are used to characterize the metabolic activity of the cells [23,40]. For comparison, the acidification rates for each cycle were calculated from the first derivative of pot(t) after *t* using the fitted time constants τ:

(4) $$\frac{d\left(pot\left(t\right)\right)}{dt}=\frac{pot_{\text{∆}}}{\tau }\left(e^{-\frac{\text{t}}{\tau }}\right)$$

An advantage of this slightly laborious way of calculation is that it compensates for the drift in the initial potentials of the measuring cycles. The drifts were roughly synchronous in the control and cell experiments (Figure 10). For consecutive measuring cycles, decreasing time constants τ were obtained from Equation (3). This was the main reason for the increasing initial values of the acidification rates of 9.6, 20.3, 28.0, and 31.8 mV/h in the four pump cycles. After 20 h the acidification rates remained largely stable.

### 3.6. The multi-Parameter Problem

Respiration and acidification rates in microfluidic systems depend on the cell number, proliferation status, *etc.* Under aerobic culture conditions, the two rates should be roughly proportional because the main source of carbon dioxide is the metabolism of glucose. In aqueous media, carbon dioxide is transformed into carbonate, which in turn leads to a decrease in the medium’s pH. Nevertheless, in practice, the correlation will be influenced by the buffering capacities of medium substances and the cells themselves. The oxygen-pH correlation may even be decoupled by metabolic mechanisms [23]. For example, a decrease in the oxygen concentration would favor anaerobic fermentation, resulting in medium acidification by lactate.

For an improved description of the cell physiology in *in vitro* systems by biochemical or cell-physiological models, a greater variety of different sensors is desirable, *i.e.*, for detecting metabolic rates or the physiological cell state. Sensors for glucose or lactate are already available [25,48]. Their data could help detecting alterations in anaerobic biochemical pathways, for example. In our system, the corresponding time constants for pH and oxygen concentration changes differ greatly from the time constants of IDES measurements. Generally, future cell culture systems should detect the interplay of these “short term” (pH, oxygen) and “long term” parameters, such as cell adhesion and proliferation as accessed by IDES. The relatively crude IDES data, may be supported by the fluorescence imaging of stress fibers, for example [19,27,49]. Additional sensors for specific cell markers or released metabolites, e.g., alkaline phosphatase or the bone morphogenetic proteins of osteoblast cell lines would be desirable for a more detailed picture of the interplay of multiple cell species, which resemble the complex biotransformation processes, e.g., in mammalian organisms (compare to ADME: “absorption, distribution, metabolism, and excretion”) [50]. Such investigations require co-culture systems with more measurable parameters. Nevertheless, a conflict between those parameters which can be accessed by existing sensors and those considered in systems biology models are likely to impose a problem in future microfluidic *in vitro* systems for the replacement of animal experiments.

## 4. Conclusions

Thin-film Pt sensors on the planar glass-substrate of cell-culture chips allow for the electric registration of physiologically relevant parameters, such as O_2_, pH, and cell adhesion [6].While thick Si_3_N_4_ layers in the µm range may serve as insulation for on-chip connectors, layers in the 20–100 nm range showed good pH sensitivity with fast response times. The sufficiently good cell-culture properties of the Si_3_N_4_ surfaces may further be improved by surface modification, e.g., molecular coatings. While the slopes and linearity of our pH sensors were lower than those of ISFET sensors, their robust structures do permit the easy sterilization and reuse of the chips. Our long-term experience with similar sensor structures showed no noticeable sensor failures; even after a dozen sterilization procedures each of which was followed by approx. two weeks of cell-culture [19]. This is a much better stability than observed with commercial silicon chips [40]. These chip properties combined with polycarbonate as a more suitable material for future system bodies permit for the sterilization of the assembled microfluidic systems with standard procedures (121°C, 30 min) prior to cell seeding. In addition, the simple construction and robustness were of clear advantage for the oxygen sensors.

Measurements with different sensor geometries have suggested that increasing sensor areas improve the sensitivity at the expense of an increased oxygen attrition. Generally, the same data recording rates can be adjusted for pH and oxygen detection. Nevertheless, high recording rates would increase the oxygen sapping by the sensor, which would not be a problem in acidification measurements. 

An array of individual IDES permitted the detection of the cell distribution inside the glass-chip system, which could be crosschecked microscopically (compare to Figure 5). Furthermore, we found that the method of data refinement was more important for the determination of cell-proliferation parameters than the pitches of the IDES sensors.

The sensor data can be used for the controlled supply of the system with nourishing substances or the stabilization of the conditions according to the demands of the cell culture. Future designs may also feature modified sensors for other compounds, which may be based on the direct optical, fluorescence [50,51] or electric detection with potentiometric, amperometric, and impedance principles [44,52,53,54].

Even though a high oxygen supply may be desirable in some cell cultures, the sensitive detection of respiration rates will require significant oxygen concentration changes. In the present system, high cell densities were required for a significant reduction of the oxygen level. The reason was the high diffusion coefficient of oxygen in PDMS (5.0 × 10^−5^ cm^2^/s at 38°C), resulting in a high oxygen feeding through the PDMS body parts [44]. This effect did not allow us to correlate the respiration with the acidification rates of the medium. Apart from the insufficient control of the oxygen supply, the sensitivity of our current setup was higher in detecting pH than oxygen concentration changes (please compare scatters in the measuring curves of Figure 7 and Figure 9).

Existing sensorized cell-chip systems are operated in a “cell-culturing mode” with large trough volumes of buffered medium and unlimited oxygen supply (open multi well plates or cell culture troughs) or in a “measuring mode” with small, confined volumes of unbuffered medium and limited oxygen supply [18,32,55]. In the latter mode, the medium exchange times are short (approx. 5 min), leading to an improved temporal parameter resolution. In the present system, we used a compromise between low exchange rates and temporal resolution.

One clear advantage of our system is the bonus of sterile conditions in a simple incubator. In order to increase the temporal resolution in future self-contained sterile systems, a small cell culture volume can be flange-mounted to a larger on‑chip reservoir [56]. In the systems, integrated ETµPs allow for high exchange rates between the on-chip reservoir and the culture volume, while the rate for the medium exchange between the on-chip and external reservoirs will be low. The on‑chip reservoir will be accessible through special fluidic ports for the administration of substances. This approach will reduce the odds for the appearance of gas bubbles, which caused problems in our current system at cell-culture times longer than one week. Already in the present system, the integrated ETµPs permitted to circulate the medium throughout the fully closed system. In future systems, ETµPs will allow for overcoming the diffusion limits for the cellular supply with oxygen and nourishing molecules, permit catabolites to be drawn out, as well as distribute substances to be tested in the cell culture. Their high‑frequency driving signals help prevent electrolytic processes at the electrodes. Optional passivation layers separate biological media and cells from the metallic surfaces [35].

Our new concept of cell monitoring in combination with flexible PDMS fluidic structures permits new experimental approaches such as the co-culture of multiple cell lines, the physical stimulation of cells, e.g., osteoblasts, or 3D-scaffolding. In principle, the 2D-limitations of planar cell-culturing systems can be overcome by sandwiching PDMS and chip layers with vertical microfluidic channels. For such systems, integrated sensors and ETµPs will be a must because stacking will reduce the microscopic observability [56,58].

The development of microfluidic cell culture systems with integrated sensors may help paving the way towards animal replacement methods for the investigation of complex biotransformation processes (ADME [59]). Such systems may feature separated cell-culture compartments with controlled medium exchange, e.g., driven by micro-pumps.
